# Epitope unmasking in vulvovaginal candidiasis is associated with hyphal growth and neutrophilic infiltration

**DOI:** 10.1371/journal.pone.0201436

**Published:** 2018-07-31

**Authors:** Eva Pericolini, Stefano Perito, Anna Castagnoli, Elena Gabrielli, Antonella Mencacci, Elisabetta Blasi, Anna Vecchiarelli, Robert T. Wheeler

**Affiliations:** 1 Department of Medicine, University of Perugia, Perugia, Italy; 2 Department of Diagnostic, Clinical and Public Health Medicine, University of Modena and Reggio Emilia, Modena, Italy; 3 School of Specialization in Microbiology and Virology, University of Modena and Reggio Emilia, Modena, Italy; 4 Department of Molecular and Biomedical Sciences, University of Maine, Orono, Maine, United States of America; 5 Graduate School of Biomedical Sciences and Engineering, University of Maine, Orono, Maine, United States of America; Louisiana State University, UNITED STATES

## Abstract

Vaginal candidiasis is a common disorder in women of childbearing age, caused primarily by the dimorphic fungus *Candida albicans*. Since *C*. *albicans* is a normal commensal of the vaginal mucosa, a long-standing question is how the fungus switches from being a harmless commensal to a virulent pathogen. Work with human subjects and in mouse disease models suggests that host inflammatory processes drive the onset of symptomatic infection. Fungal cell wall molecules can induce inflammation through activation of epithelial and immune receptors that trigger pro-inflammatory cytokines and chemokines, but pathogenic fungi can evade recognition by masking these molecules. Knowledge about which cell wall epitopes are available for immune recognition during human infection could implicate specific ligands and receptors in the symptoms of vaginal candidiasis. To address this important gap, we directly probed the surface of fungi present in fresh vaginal samples obtained both from women with symptomatic *Candida* vaginitis and from women that are colonized but asymptomatic. We find that the pro-inflammatory cell wall polysaccharide β-glucan is largely masked from immune recognition, especially on yeast. It is only exposed on a small percentage of hyphal cells, where it tends to co-localize with enhanced levels of chitin. Enhanced β-glucan availability is only found in symptomatic patients with strong neutrophil infiltration, implicating neutrophils as a possible driver of these cell wall changes. This is especially interesting because neutrophils were recently shown to be necessary and sufficient to provoke enhanced β-glucan exposure in *C*. *albicans*, accompanied by elevated immune responses. Taken together, our data suggest that the architecture of *C*. *albicans* cell wall can be altered by environmental stress during vaginal candidiasis.

## Introduction

*C*. *albicans* causes both mucosal and disseminated disease, but vaginitis is the only disease it causes in healthy adults [1]. Acute vulvovaginal candidiasis (VVC) is estimated to affect two-thirds of all women during their lifetimes, while recurrent VVC (RVVC) is more severe but less common. Unfortunately, we still know little about why some women suffer from VVC or RVVC and others do not [2–5]. Both VVC and RVVC are usually caused by *C*. *albicans*, but can be caused by *C*. *glabrata*, *C*. *krusei*, and many other species [2, 4]. Therapy for VVC relies on a variety of azoles, which are of limited use due to concerns about the use of fluconazole in pregnancy [6–8].

Vaginitis symptoms from *C*. *albicans* infection are thought to stem from host immune responses rather than pathogen-mediated damage [9]. Support for this idea comes from a unique human challenge study that found that neutrophilic inflammation, rather than fungal burden, was the greatest predictor of symptomatic infection in otherwise healthy women [10]. Host inflammatory processes can be triggered by fungal cell wall molecules and proteins that interact with receptors of innate immune and epithelial cells [11–15]. Despite their well-recognized capacity to stimulate inflammation, we still understand little about the fungal cell wall ligands that are actually available for immune recognition during vaginal infection, especially during human infection.

Pattern recognition of fungal cell wall components is limited by pathogens that regulate the availability of specific epitopes for interaction with immune receptors [16, 17]. *C*. *albicans* and other fungi can mask cell wall β-glucan and chitin from recognition by Dectin-1 and other PRRs, but it is unknown if this mechanism of evasion is used in human infection [16]. Some *in vivo* and *in vitro* models suggest that β-glucan is largely masked [18, 19], but other *in vitro* experiments have documented some β-glucan exposure during epithelial infection [20]. These differences among models may arise because each model presents a unique subset of signals that regulate exposure of cell wall epitopes, including environmental cues, stresses, and immune-triggered cell wall remodeling [17]. This is especially intriguing for VVC, where *in vitro*-validated cues for masking (lactate) and unmasking (low pH) are both expected to be present [21, 22]. It is difficult to exactly replicate the environmental conditions during human infection *in vitro*, especially because they are likely to be dynamic and to depend on infection progression in individual patients.

Clinical studies of epitope exposure are technically and logistically challenging because epitope exposure is sensitive to fixation conditions and is ideally studied in directly-obtained samples. Nevertheless, such work could reveal important information about host-pathogen interaction during human disease and point to which experimental model(s) would be best for the study of epitope recognition during infection. We have addressed the technical issues associated with such a clinical study by probing the surface of *C*. *albicans* that was freshly isolated from vaginitis patients. In this first study of epitope exposure in human infection, we find only rare sites of β-glucan exposure on hyphal cells. These cases of β-glucan unmasking are often associated with chitin deposition, are found only in the presence of strong neutrophilic infiltrates, and correlate with evidence of neutrophil extracellular traps. These data suggest both that *C*. *albicans* masks cell wall components from immune recognition *in vivo* and that neutrophils may drive some limited level of unmasking during human infection.

## Materials and methods

### Ethics statement

All women signed an informed consent in accordance with the Declaration of Helsinki. Local Ethical Committee CEAS (Comitato Etico delle Aziende Sanitarie, Umbria, Italy) approval was received for the whole study (VAG1 n. 2652/15) on February 1, 2016.

### Subjects

Thirty *Candida*-positive non-diabetic women, 19 to 53 years old, attending the microbiological diagnostic service of the University Hospital Santa Maria della Misericordia, Perugia (Italy) over the period from February 2016 to April 2017 were consecutively enrolled in this study. Enrollment and consent was done as described [23]. This included both those who attended for both routine screening and those with symptoms of vaginal disease. Prior to enrollment, each subject answered a questionnaire indicating their health status and current symptoms of vaginal disease. None of the patients was taking any treatments for symptoms at the time of enrollment. A case of VVC due to *Candida* was defined as the presence of at least two of the following signs and symptoms: vaginal discharge, itching, burning, dyspareunia, and *Candida* isolation from the vaginal sample. None of the recruited women had RVVC, as indicated by the absence of documented or subject-reported, repeated VVC episodes per year. All women signed an informed consent in accordance with the Declaration of Helsinki. Local Ethical Committee CEAS (Comitato Etico delle Aziende Sanitarie, Umbria, Italy) approval was received for the whole study (VAG1 n. 2652/15).

### Neutrophil infiltration scoring, pH analysis and bacterial identification

Vaginal swabs were taken and soaked in 1 ml of saline, as described in [23]. To measure pH, a vaginal swab was taken from each participant and soaked in 1 ml of saline. The pH was measured by pH-Fix strips (Macherey-Nagel GmbH & Co. KG, Germany). The morphotypic evaluation of vaginal microbiota was performed by the observation of a fresh preparation of vaginal fluid or by Gram staining method under the light microscope. In particular, the presence or absence of *Lactobacilli* was verified. After sampling, the vaginal swab was plated on Tryptic Soy Agar II with 5% sheep blood or on *Gardnerella* Selective Agar with 5% human blood or on Columbia Agar with 5% Sheep Blood (all from Becton Dickinson) to test the vaginal colonization by Lactobacilli, *G*. *vaginalis* or *S*. *agalactiae* (Group B Streptococcal or GBS), respectively. Samples were examined under light microscope (Olympus, Milan, Italy) to evaluate the presence of neutrophils (PMN) by their morphology after staining with Papanicolaou technique, as described in [10, 23]. The number of PMN was counted in four fields at x400 magnification and expressed as average number of PMN/field. The PMN score was graded on a scale from 0 to 2: 0, 0 PMN/field; 1, 1 to 10 PMN/field; 2, 11 to 40 PMN/field as previously described [23]. This gradation allowed for semi-quantitative analysis of neutrophil numbers while limiting burden on attending physicians. *Candida*-positive vaginal samples were categorized as: no or low neutrophil infiltration (LI; score ≤ 1) or high neutrophil infiltration (HI; score = 2). This cohort included women who were *C*. *albicans*-positive with high neutrophil infiltration (HI; n = 15), *C*. *albicans*-positive with low-no neutrophil infiltration (LI; n = 7), non-*albicans Candida* (NAC) with high neutrophil infiltration (HI; n = 2), and NAC with no or low neutrophil infiltration (LI; n = 6). pH was measured in vaginal samples.

### Sample collection and species identification

A separate vaginal swab was taken from each subject and soaked in 2 ml of PBS. After sampling, the vaginal swab was plated on CHROMagar^™^ Candida (VWR International p.b.i., Milan, Italy) at 37°C for 48 hours to evaluate the vaginal colonization by *C*. *albicans*. The presence of *C*. *albicans* was confirmed by MALDI-TOF using the VITEK MS system (Biomérieux S.A. France). Subsequently, the vaginal fluid was centrifuged at 3,000 x rcf for 5 min. The cellular fraction in the pellet was resuspended and half was used for live staining.

### Live staining of the cellular fraction of vaginal swabs

Vaginal swabs were processed within 5 hours (hr) of collection, without fixation, similar to processing for *ex vivo* fluorescence [24]. Previous work suggests that fixation can alter cell surface characteristics (Wheeler et al. 2006), so this was avoided at all costs. Initial tests of staining *C*. *albicans* from vaginal swabs after 1 hr, 5 hr, and 18 hr post-collection, with storage at room temperature in saline, showed no differences in staining levels. However, to limit any artifacts of storage, the stainings were all performed within 5 hr of collection. The cellular fraction of the vaginal swab was washed three times in phosphate buffered saline (PBS), with a 15-second spin at maximum speed (15,000 x rcf) in the microcentrifuge (Eppendorf 5415C), decanting of the supernatant, resuspension of the pellet in the residual volume, then addition of 500 μl of ice-cold PBS. Following the initial wash steps and prior to adding the staining reagents, cells were resuspended in 100 μl of PBS + 1% BSA by flicking the pellet, 1 x 10^6^ KAH3-EGFP cells was added as a positive internal control, and the sample was briefly incubated on ice for 5 minutes to block non-specific staining. Once washed and blocked, staining reagents were added: sDectin-1-Fc (1.25:100 from 1.4 μg/microliter), Calcofluor white (5:100 from 100 μg /ml stock) and Sytox Green (0.25:100 from 5 mM stock). The sDectin-1-Fc was purified from HEK293T cells as described [25] then labeled directly with Alexafluor 555. To directly label the sDectin-1-Fc, 220 micrograms of purified protein (100 μl in PBS + 45% glycerol) was mixed with 100 μg of Alexafluor 555-succinimidyl ester (lyophilized; Thermo Fisher) and 10 μl of 1 M NaCO_3_ for a final pH of 8.5, incubated at room temperature for one hour, run through a gel filtration spin column pre-equilibrated with PBS (G-25, GE Healthcare), and then adjusted to 45% glycerol in PBS. Calcofluor white (Fluorescent Brightener 28 F3543; Sigma-Aldrich) was dissolved in water. Sytox Green (Thermo Fisher) was dissolved in dimethylsulfoxide. The KAH3-EGFP strain can be distinguished from yeast in the vaginal swab sample because it expresses cytoplasmic EGFP, and it has previously been described to have enhanced β-glucan exposure and sDectin-1 staining [19]. Then, the staining reagents were added, and the cells were further incubated for 30 minutes on ice. Cells were then washed three times in one milliliter of PBS as above and were resuspended in 50 μl. Ten μl of sample were spread in a line on a microscope slide and covered with a #1 coverslip (24 mm x 60 mm) for imaging.

### Microscopic analysis

**Fig 1:**
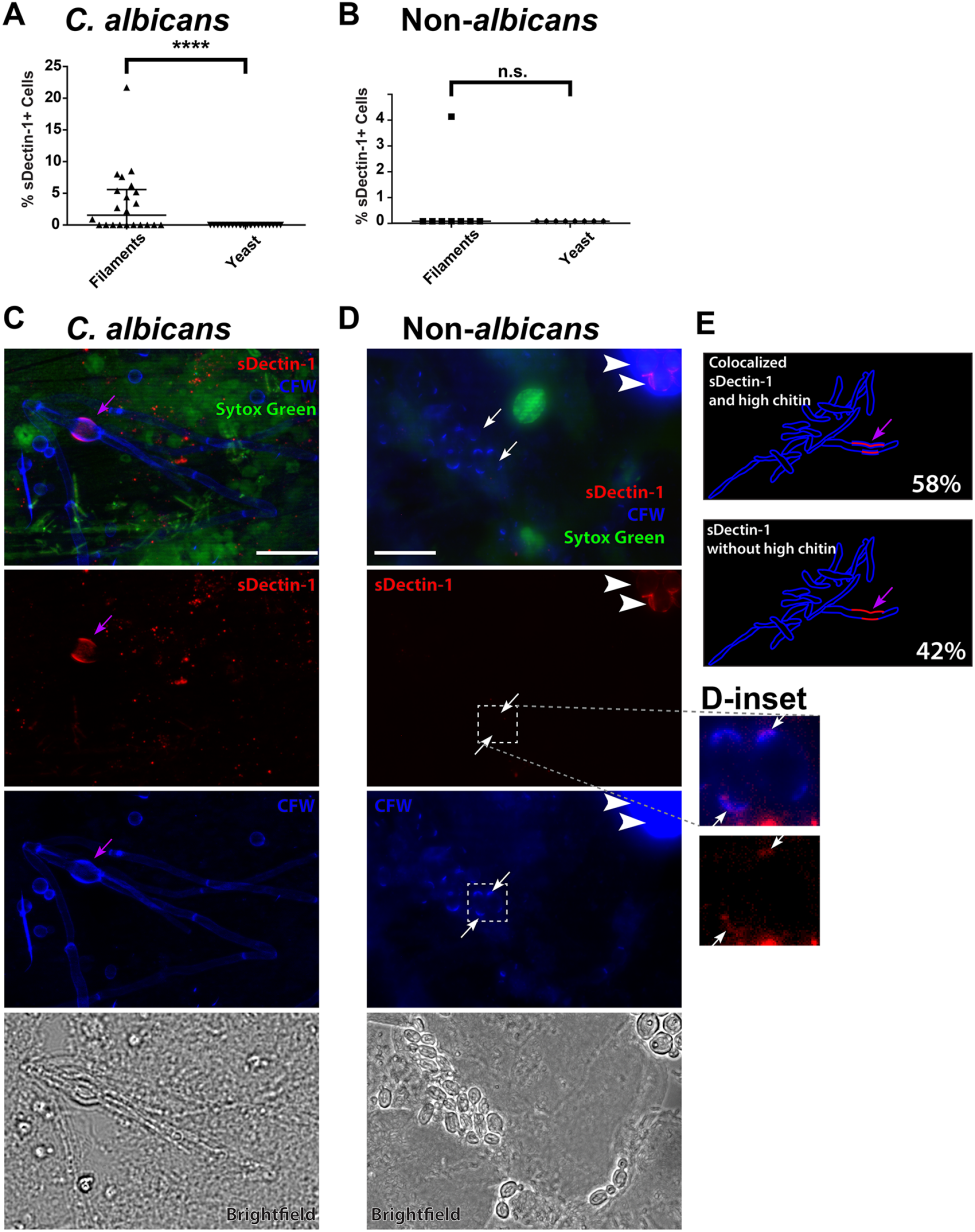
Enhanced Dectin-1 recognition is filament-specific and co-localizes with enhanced chitin deposition. Vaginal swab samples were stained with sDectin-1-Fc (sDectin-1; red), Calcofluor white (CFW; blue; outline of fungi for morphology and for identification of sites of increased chitin deposition), and Sytox Green (green; stains only extracellular DNA and DNA inside cells with compromised membranes). The EGFP-expressing strain KAH3-EGFP was spiked in before staining as a positive control. Several 40x fields (8–16 per sample) were imaged and scored for fungal morphology, sites of sDectin-1 staining, co-localization of sDectin-1 staining and increased CFW staining, and presence of extracellular DNA. (A) In samples from *C*. *albicans*-infected patients, only filaments were found with enhanced sDectin-1 staining. (B) In samples from patients infected with non-*albicans Candida* or a mix of *C*. *albicans* and non-*albicans Candida*, only filaments were stained with sDectin-1. The only sample with any sDectin-1+ cells was a mix of *C*. *albicans* and *C*. *norvegensis* (Sample #SP-97366). The difference in frequency of staining is not significantly different between filaments and yeast (p >0.9999), because there was virtually no staining of either morphology. (C) Representative field from *C*. *albicans*-infection sample (#SP-66117). A single filament segment that is swollen (purple arrow) has high levels of sDectin-1 and CFW staining. The top image is a three-color overlay and the images below separate the sDectin-1 and CFW. (D) Representative field from a non-*albicans Candida* infection sample (#SP-12622). Most of the fungi are yeast from the infected patient without high levels of sDectin-1 or CFW staining (white arrows). In the upper right is a cluster of cells from the spiked-in control KAH3-EGFP (white arrowheads) with enhanced sDectin-1 and CFW staining. The CFW is overexposed to visualize the weakly-staining *C*. *krusei* cells from the patient, but the cell outlines can be clearly seen in the sDectin-1 staining image in red. (E) Schematic to illustrate that the majority of sites (58%) with enhanced sDectin-1 staining also had increased chitin deposition. Images are maximum projections of 6 slices (J) or 5 slices (K). Scalebar = 20 μm throughout. Statistics used for (H) and (I) were Mann-Whitney non-parametric tests. Significance throughout the figure is indicated with: n.s. p > 0.05; * p < 0.05; ** p < 0.01; *** p < 0.001; **** p < 0.0001.

Samples were imaged with a Zeiss Axiobserver Z1 microscope and Axiocam MRm camera using DAPI, FITC, and Rhodamine filter sets, using a Plan Neofluor 40x 0.75 N.A. air objective and Zeiss Axiovision software. Z-stacks were processed in ImageJ by creating maximum projections and then scored as follows. There were rare broken *C*. *albicans* filaments (<3% of the total) that were excluded from analysis because it was not known if the breakage was due to immune activity during infection response or due to the shear stress associated with resuspending the cell pellet during staining. sDectin-1-positive cells and hyphal segments were identified based on having staining above background. Counts were pooled for all fields imaged of a given sample. Morphology was scored by counting all elongated cells as part of filaments, either pseudohyphae or hyphae, using Calcofluor white staining to identify septae and thus filament segments. Counts were pooled for all fields imaged of a given sample. A field with any extracellular, diffuse Sytox Green staining was scored as positive for eDNA, and the percentage of all imaged fields with any eDNA was calculated. Maximum projections of representative images were further processed, with equal adjustments for all images, in Photoshop and Illustrator to add notations and scalebars. Where noted, images were enhanced to emphasize a specific finding, such as the extremely weak but noticeable sDectin-1 staining of bud scars on yeast shown in the inset for Fig 1.

### Statistical analysis

All statistics were performed in Graphpad 6 (Prism). Mann-Whitney and Spearman correlation tests were used as non-parametric tests, as indicated in Figure Legends. Significance throughout the figures is indicated with: n.s. p > 0.05; * p < 0.05; ** p < 0.01; *** p < 0.001; **** p < 0.0001.

## Results

The innate immune system recognizes *C*. *albicans* through pattern recognition receptors that are triggered by cell wall molecules that include β-glucan [12]. *C*. *albicans* and other fungi are able to avoid recognition by reducing β-glucan availability, although it is not yet understood if any fungus evades β-glucan detection during human infection [24]. We sought to measure β-glucan exposure in fungi during vulvovaginal infection. We recruited patients from the microbiological diagnostic service of the University Hospital Santa Maria della Misericordia, including both patients that came to the clinic complaining of vaginitis and pregnant women coming for routine screening. Vaginal lavages were screened for the presence of fungi and scored for levels of neutrophilic infiltrate (HI or LI) as previously detailed [23].

Samples with fungi were processed for microbiological identification and measurement of fungal epitope exposure. Identification was made by the complementary methods of MALDI-TOF and Chromagar growth. In most cases (29/30), results were congruent. In one case (#SP-97366), more than one type of colony was identified on Chromagar, and these colonies were subsequently processed for identification by MALDI-TOF, which confirmed the Chromagar identification. The majority of cases were due to *C*. *albicans* (22/30), with occasional infection by *C*. *glabrata* (3/30), *C*. *krusei* (2/30) and others (Table 1).

**Table 1:** Staining with sDectin-1 on hyphae is associated with neutrophil infiltration and extracellular DNA.

Neutrophil Infiltration	Patient #	Fungal species	% Hyphae	% sDectin-1-positive (Hyphal)	% sDectin-1-positive (yeast)	% Fields with Extracellular DNA
High	SP-14248	*C*. *albicans*	67.3	6.35	0	100
	SP-12635	*C*. *albicans*	53.7	0.27	0	100
	SP-12522	*C*. *albicans*	82.7	7.2	0	100
	SP-11266	*C*. *albicans*	32.7	1.4	0	100
	SP-01417	*C*. *albicans*	88.1	5	0	57.1
	SP-97296	*C*. *albicans*	64.4	3.3	0	100
	SP-17001	*C*. *albicans*	0	0	0	14.2
	SP-94976	*C*. *albicans*	48.6	5.7	0	100
	SP-15980	*C*. *albicans*	66.3	21.7	0	100
	SP-00703	*C*. *albicans*	76.4	8.4	0	100
	SP-99758	*C*. *albicans*	73.2	10.3	0	100
	SP-01887	*C*. *albicans*	83.7	4.4	0	100
	SP-63354	*C*. *albicans*	86.4	17.3	0	100
	SP-50711	*C*. *albicans*	22.4	10.1	0	100
	SP-66117	*C*. *albicans*	31.4	9.3	0	100
Low or None	SP-18387	*C*. *albicans*	22.5	0	0	100
	SP-15231	*C*. *albicans*	9.5	0	0	100
	SP-14314	*C*. *albicans*	1.2	0	0	12.5
	SP-12753	*C*. *albicans*	23	0	0	14.2
	SP-00503	*C*. *albicans*	0	0	0	42.8
	SP-16884	*C*. *albicans*	11.9	0	0	85.7
	SP-16850	*C*. *albicans*	27.8	0	0	0
High	SP-12622	*C*. *krusei*	0	0	0	83.3
	SP-97366^&^	*C*. *albicans/ C*. *norvegensis*	81.3	3.3	0	83.3
Low or None	SP-11225	*C*. *glabrata*	0	0	0	0
	SP-13835	*C*. *glabrata*	0	0	0	42.8
	SP-15936	*C*. *glabrata*	0	0	0	33.3
	SP-15949	*C*. *krusei*	0	0	0	22.2
	SP-15194	*C*. *guilliermondii*	1.6*	0	0	100
	SP-90841	*S*. *cerevisiae*	0	0	0	0

Top samples are *C*. *albicans* only. Bottom set are mixed or only non-*albicans Candida*.

^&^ Sample SP-97366 was mixed approximately 50–50 with *C*. *norvegensis* (identified by MALDI-TOF; white colonies on CHROMagar) and *C*. *albicans* (identified by green colonies on CHROMagar). Based on known morphotypes of these species, it is likely that all the hyphae in this sample were *C*. *albicans*.

* Sample SP-15194 had filaments but these were only pseudohyphae.

Epitope exposure was measured using an *ex vivo* fluorescence protocol modified from that used for probing live fungi isolated directly from infected mouse organs [24]. In this fluorescence microscopy assay, we used soluble Dectin-1-Fc (sDectin-1) to monitor β-glucan exposure, the chitin-staining dye Calcofluor white (CFW) to monitor chitin deposition and fungal morphology, and the membrane-impermeable dye Sytox Green to identify dead fungi and stain extracellular DNA. We note that CFW staining is a good quantitative measure of chitin levels in *C*. *albicans* [26], but has been shown to bind to other polysaccharides such as cellulose that have not been found in *C*. *albicans* cell walls [27]. Due to the low overall frequency of sDectin-1-positive cells, an EGFP-expressing mutant of *C*. *albicans* (KAH3) with moderate levels of β-glucan exposure [18] was spiked in as a positive control to ensure even staining from sample to sample (S1 Fig).

Only a small proportion of live fungi from vaginal lavages stained strongly with sDectin-1, and this varied significantly from patient to patient (Table 1, Fig 1A). For *C*. *albicans*, sDectin-1 staining was seen exclusively in hyphal and pseudo-hyphal filaments, and only a small percentage of those cells (Fig 1A and 1C). No high-level sDectin-1 staining was seen in other species, including *C*. *glabrata* and *C*. *krusei*, although very weak sDectin-1 staining was seen occasionally in these yeast at bud scars, as has been previously reported (Fig 1B and 1D & D-inset; [18, 28]). Note that the contrast on these inset images had to be extremely enhanced to bring out even this faint staining. Nearly all of the sites of sDectin-1 reactivity in live cells were in the lateral wall of filaments, and the Dectin-1-positive cell segments were often swollen. Interestingly, the majority of Dectin-1-positive sites had elevated levels of CFW staining (Fig 1C–1E and S1 Fig). Some dead fungal cells had high labeling with sDectin-1 at the ends of broken filaments that may have been broken during the collection and staining procedure (S2 Fig). Overall, sDectin-1 reactivity was infrequent, was only seen in *C*. *albicans* hyphae, and was associated with increased CFW staining that suggests high chitin deposition.

**Fig 2:**
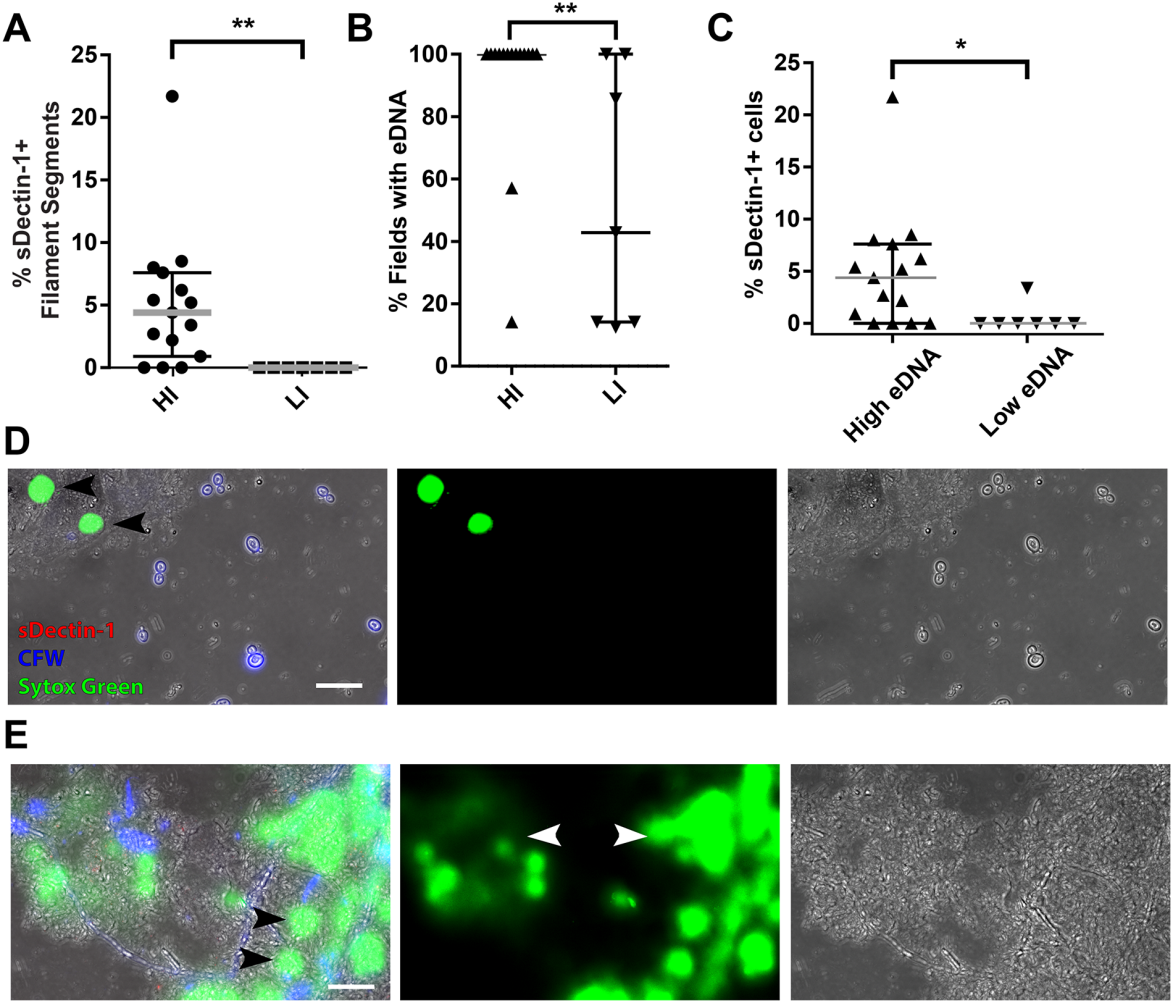
Neutrophil infiltration and extracellular DNA are closely associated with enhanced sDectin-1 staining. Vaginal swab samples with *C*. *albicans* were scored by level of neutrophil infiltration, then stained and imaged as in Fig 1. (A) Samples with high levels of neutrophil infiltration had sDectin-1-positive filaments, while those with low levels of infiltration had none. (B) Samples with high infiltration had a significantly higher level of extracellular DNA (eDNA), as imaged using Sytox Green. (C) Samples with high levels of eDNA also had high percentages of sDectin-1-positive cells. Samples with eDNA in every field were categorized as High eDNA, samples for which <100% of fields had eDNA were categorized as Low eDNA. (D) Representative field of a sample (#SP-14314) with no fields containing eDNA, no hyphae, and no sDectin-1-positive cells. Black arrowheads indicate nuclei from epithelial cells in the lavage. Image is maximum projection of 5 z-slices, created by ImageJ. (E) Representative field of a sample (#SP-12522) with high levels of eDNA, hyphae, and sDectin-1 positive cells. Black arrowheads indicate epithelial nuclei and white arrowheads indicate areas of diffuse Sytox Green staining of eDNA. Image is maximum projection of 10 z-slices, created by ImageJ. Scalebar = 20 μm. Statistics used in (A-C) were Mann-Whitney non-parametric tests. Significance throughout the figure is indicated with * p < 0.05, ** p < 0.01, *** p < 0.001.

Because co-localization of enhanced sDectin-1 reactivity and chitin deposition can be triggered by neutrophil-mediated damage of *C*. *albicans* hyphae [24], we suspected that these phenotypes were caused by neutrophilic attack. To test for association between neutrophilic attack and sDectin-1 recognition in VVC, we compared the prevalence of sDectin-1-staining filament segments in patient samples depending on neutrophilic infiltration. Strikingly, β-glucan exposure was only found in samples from patients with high neutrophilic infiltration (Fig 2A). Neutrophil-triggered β-glucan exposure and chitin deposition in *C*. *albicans* filaments can be blocked *in vitro* by eliminating neutrophil extracellular traps (NETs) that contain extracellular DNA (eDNA; [24]). To test for a connection between eDNA and sDectin-1 staining, we scored vaginitis samples from each patient for eDNA by staining with Sytox green; Dectin-1-labeled fungi were only found in those samples with high prevalence of extracellular DNA (Fig 2B). Furthermore, there is a significantly higher frequency of sDectin-1-positive cells in samples with high levels of eDNA (100% of fields with eDNA; Fig 2C). Representative images illustrate both the stained nuclei of shed epithelial cells (Fig 2D) and the diffuse staining typically associated with extracellular traps (Fig 2E). Unfortunately, with the reagents we used it is not possible to tell if there are bacterial organisms also associated with the eDNA. Although these data are consistent with the presence of NETs, a more focused study would be required to unambiguously identify these structures as NETs.

Vaginitis has been suggested to be associated with microbial dysbiosis, so vaginal swabs were also scored clinically for the presence of *Lactobacilli* and other bacteria. Lactobacilli were found in all samples, as has been seen before [29], suggesting that infection is not due to the loss of these bacteria in our patient population (Table 2). *Lactobacilli* can reduce pH by producing lactic acid, and elevated vaginal pH has been suggested to predispose for VVC, so pH was measured in vaginal samples. As expected, we found that all measured samples had an acidic pH range of 4–5, consistent with the ubiquitous presence of *Lactobacilli* in all patients (Table 2). To further test a possible relationship between unmasking and low pH, we compared the frequency of Dectin-1-positive cells in pH 4 vs. pH 5 samples and found no significant correlation (Mann-Whitney; p = 0.3723).

**Table 2:** *Lactobacilli* are found in most samples and are not associated with neutrophil infiltration or lack of sDectin-1+ cells.

Patient #	Fungi	Neutro	sDectin-1+	Bacteria	*Lacto*.	*G*. *vag*.	GBS	Pregnant	pH	Sympto-matic?
SP-14248	*C*. *a*.	**High**	6.35	+	+	-	+	N	4	Y
SP-12635	*C*. *a*.	**High**	0.27	+	-	+	+	N	n.a.	Y
SP-12522	*C*. *a*.	**High**	7.2	+	+	-	-	N	4	Y
SP-11266	*C*. *a*.	**High**	1.4	+	+	-	-	N	n.a.	Y
SP-01417	*C*. *a*.	**High**	5	+	+	-	-	N	n.a.	Y
SP-97296	*C*. *a*.	**High**	3.3	+	+	-	-	N	n.a.	Y
SP-17001	*C*. *a*.	**High**	0	+	-	+	-	Y	5	Y
SP-94976	*C*. *a*.	**High**	5.7	+	+	-	-	Y	n.a.	Y
SP-15980	*C*. *a*.	**High**	21.7	+	+	-	+	Y	n.a.	Y
SP-00703	*C*. *a*.	**High**	8.4	+	+	+	-	Y	5	Y
SP-99758	*C*. *a*.	**High**	10.3	+	+	-	-	Y	5	Y
SP-01887	*C*. *a*.	**High**	4.4	+	+	-	-	Y	5	Y
SP-63354	*C*. *a*.	**High**	17.3	+	+	-	-	Y	n.a.	Y
SP-50711	*C*. *a*.	**High**	10.1	+	+	-	-	Y	n.a.	Y
SP-66117	*C*. *a*.	**High**	9.3	+	+	-	-	Y	n.a.	Y
SP-18387	*C*. *a*.	**Low**	0	+	+	-	-	N	n.a.	N
SP-15231	*C*. *a*.	**Low**	0	+	+	-	+	N	4	N
SP-14314	*C*. *a*.	**Low**	0	+	+	-	-	N	4	N
SP-12753	*C*. *a*.	**Low**	0	+	+	-	-	N	5	N
SP-00503	*C*. *a*.	**Low**	0	+	+	-	-	N	n.a.	N
SP-16884	*C*. *a*.	**Low**	0	+	+	-	-	Y	4	N
SP-16850	*C*. *a*.	**Low**	0	+	+	-	+	Y	4	N
SP-12622	*C*. *k*.	**High**	0	+	+	-	-	N	4.5	Y
SP-97366	*C*. *a*.*/ C*. *n*.^&^	**High**	3.3	+	+	-	-	N	5	Y
SP-11225	*C*. *gl*.	**Low**	0	+	+	-	-	N	n.a.	N
SP-13835	*C*. *gl*.	**Low**	0	+	+	-	-	N	4	N
SP-15936	*C*. *gl*.	**Low**	0	+	+	-	-	N	4	N
SP-15949	*C*. *k*.	**Low**	0	+	+	-	-	N	5	N
SP-15194	*C*. *guil*.*	**Low**	0	+	+	-	-	N	5	N
SP-90841	*S*. *c*.	**Low**	0	+	-	-	+	Y	n.a.	N

Top samples are *C*. *albicans* only. Bottom set are mixed or only non-*albicans Candida*.

^&^ Sample SP-97366 was mixed with *C*. *norvegensis* (identified by MALDI-TOF) and *C*. *albicans* (identified by CHROMagar). Based on known morphotypes of these species, it is likely that all the hyphae in this sample were *C*. *albicans*.

* Sample SP-15194 had filaments but these were only pseudohyphae. Abbreviations: *C*.*a*. (*C*. *albicans*), *C*.*n*. (*C*. *norvegensis*), *C*. *gl*. (*C*. *glabrata*), *C*. *k*. (*C*. *krusei*), *C*. *guil* (*C*. *guilliermondii*), *S*. *c*. (*Saccharomyces cerevisiae*). n.a. = data not available for this sample.

## Discussion

Interaction of *Candida* with host cells depends on the presence of host receptors and the availability of fungal ligands, but we know little about ligand exposure during human infection. In this work, we found that β-glucan, a pro-inflammatory ligand for innate immune receptors, is largely masked from recognition in fungi during acute vulvovaginal candidiasis in women. However, strong neutrophil infiltration is associated with the presence of some hyphal *C*. *albicans* cells with enhanced recognition by the Dectin-1 β-glucan receptor. These sites resemble areas of cell wall remodeling that are found *in vivo* during murine invasive candidiasis and are triggered by neutrophil attack on hyphae [24]. In contrast, infecting *C*. *albicans* yeast and non-*albicans Candida* species without hyphae are not associated with strong neutrophilic infiltrates and do not have localized areas of β-glucan exposure.

Previous *in vivo* and *in vitro* studies of *C*. *albicans* that have shown that environmental stresses, drugs, and immune attack can all trigger cell wall remodeling that alters surface epitope exposure and immune recognition [17, 30]. Consistent with these previous reports, our clinical finding of a small but reproducible percentage of hyphal cells with enhanced epitope exposure in the lateral cell wall suggests that *C*. *albicans* can and does actively remodel its cell wall during infection. The strong correlation we found between conditions of high neutrophil infiltration and the presence of sDectin-1-reactive hyphal segments suggests that neutrophils may play a role in triggering cell wall remodeling events during human infection. Neutrophilic infiltration correlated more strongly with VVC symptoms than fungal burden in an important acute challenge study [10]. Consistent with this idea, mouse VVC models suggest that neutrophils are not particularly effective at limiting fungal burden but instead drive symptomatic infection by enhancing inflammation [9, 31]. Taken in this context, the strong correlation of neutrophil infiltration with unmasking we found may only reflect an indirect relationship between neutrophil attack and unmasking. Alternatively, neutrophils may have reduced fungicidal activity in the vagina during VVC, but still have enough activity to create extracellular traps and trigger cell wall remodeling that enhances β-glucan exposure and Dectin-1 binding. Moreover, given the role of inflammatory cytokines in neutrophil recruitment, greater β-glucan exposure could play a causative role in the high infiltration that is associated with β-glucan exposed hyphal segments [32–34].

The presence of cells with strong Dectin-1 binding suggests they might drive greater Dectin-1-dependent inflammatory responses, as is the case *in vitro* [16]. However, this might not be an important driver of human inflammatory responses in VVC, because Dectin-1 mutation is not associated with altered susceptibility to RVVC [35]. Instead, Dectin-1 binding may indicate areas of enhanced β-glucan exposure that lead to greater activation of the EphA2 receptor, a newly described epithelial cell receptor for β-glucan [33]. There may also be altered exposure of other cell wall polysaccharides such as chitin, which is also exposed at greater levels in the context of β-glucan exposure [17]. If greater epitope exposure through neutrophilic attack leads to stronger inflammatory responses *in vivo*, it may be possible to limit VVC symptoms by limiting epitope unmasking.

We found that, during colonization and active vaginitis, *Candida* yeast had extremely low availability of β-glucan and only a small percentage of hyphal cells had strong β-glucan exposure. Recent work *in vitro* suggests that two different environmental cues of the vaginal milieu can signal changes in *C*. *albicans* epitope exposure and consequent immune stimulation. On one hand, the presence of lactate enhances β-glucan masking and reduces immune recognition [21]. On the other hand, acidic pH reduces β-glucan masking and increases immune responses [22]. We found *Lactobacilli* in all patients and measured acidic pH for all samples, suggesting the presence of lactate and low pH in the vaginal environment of all patients. It should, however, be noted that due to practical limits of our approved IRB protocol, our technique for measuring pH was not the most precise method possible [36]. This is in agreement with other studies that find no alteration in levels of lactic acid or pH in VVC patients [37, 38]. Our finding of only a small percentage of *C*. *albicans* filament segments with sDectin-1 reactivity suggests that the masking effects of lactate may dominate over the effects of low pH in human VVC. However, it is possible that the strains differ significantly from the clinical SC5314 strain, used in evaluation of the in vitro effects of nutritional or pH alterations, in their basic cell wall composition or response to stresses. The masking we observe *in vivo* is likely due to active fungal evasion, but there may be host molecules such as natural anti-polysaccharide antibodies that contribute to masking [32, 39]. Further *in vivo* studies focused on more precise measurement of pH, lactate and lactic acid in vaginal fluid, combined with *in vitro* studies on a wider array of vaginal isolates, are likely to be more conclusive.

Taken together with previous findings from *in vitro* and murine models, these data provide the first indication that *C*. *albicans* immune evasion by epitope masking is not completely effective during human infection. There are potential implications of this work for the treatment of VVC. First, unmasked β-glucan may provoke stronger inflammatory responses through direct binding to pro-inflammatory receptors and/or through activating complement [32–34]. Recent work suggests that patients with high neutrophilic infiltrate also have high levels of inflammatory cytokines associated with symptoms [23]. Thus, treatments that limit unmasking may also reduce inflammation-associated symptoms. Interestingly, fluconazole is effective in treatment of VVC and does not enhance β-glucan exposure in murine infection, while echinocandins can unmask β-glucan and provoke greater inflammation [18]. Studies of β-glucan exposure during treatment of patients could be informative about potential immunomodulatory effects of antifungal therapy. Second, if environment-triggered unmasking enhances inflammation, treatments targeting this process might effectively limit the symptoms of vaginitis without affecting fungal burden. This lack of inflammatory activity may be an important consideration as new therapeutics are developed against azole-resistant strains of *Candida*.

## Supporting information

S1 Fig Example of the use of the spiked-in KAH3-EGFP strain to control for the staining methodology. Vaginal swab samples were stained as described for Fig 1. In this field, there is an example of a KAH3-EGFP cell (indicated with a white arrowhead) with a green cytoplasm due to EGFP expression and high levels of chitin and sDectin-1 staining. There are also three examples of filament segments that are sDectin-1+ (indicated with purple arrows). Each of these sites also has enhanced levels of chitin staining, and one is a septum. Scalebar = 20 μm. Field is from sample #SP-12522, and is *C*. *albicans*. Image is maximum projection of 11 slices, created by ImageJ.
(PDF)

S2 Fig Examples of dead and broken filament segments with sDectin-1 staining. Vaginal swab samples were stained as described for Fig 1. In many fields, there were broken ends of filaments that were dead and excluded from analysis. In each of the two representative fields, there is one filament segment that is broken and stained with sDectin-1 (indicated with a white arrow). In panel A, the broken filament end only has sDectin-1 staining on the tip. In panel B, the broken end has sDectin-1 staining descending from the end. Note that in neither case is there enhanced chitin deposition in the cell wall with sDectin-1 staining. Scalebar = 20 μm. Fields are from sample #SP-15980, which is *C*. *albicans*. Image is maximum projection of 10 z-slices, created by ImageJ.
(PDF)
