# Supplementary figures and images for: Epitope unmasking in vulvovaginal candidiasis is associated with hyphal growth and neutrophilic infiltration
Source: PLoS One. 2018 Jul 31;13(7):e0201436. doi: 10.1371/journal.pone.0201436 (PMC6067721; doi:10.1371/journal.pone.0201436)

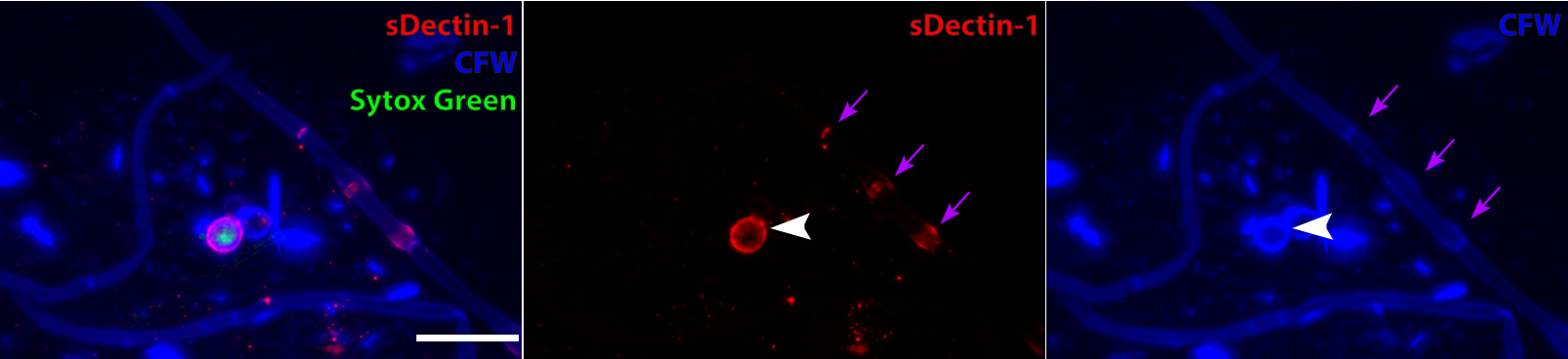

**Figure S1**

Supplement: S1 Fig — Vaginal swab samples were stained as described for Fig 1. In this field, there is an example of a KAH3-EGFP cell (indicated with a white arrowhead) with a green cytoplasm due to EGFP expression and high levels of chitin and sDectin-1 staining. There are also three examples of filament segments that are sDectin-1+ (indicated with purple arrows). Each of these sites also has enhanced levels of chitin staining, and one is a septum. Scalebar = 20 μm. Field is from sample #SP-12522, and is C. albicans. Image is maximum projection of 11 slices, created by ImageJ. (PDF) [file pone.0201436.s001.pdf]

**A**

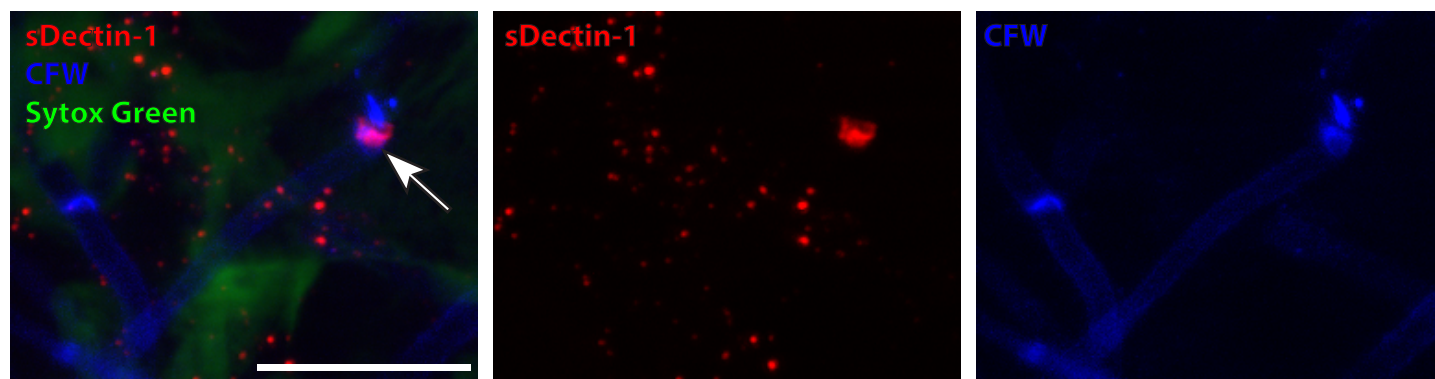

**B**

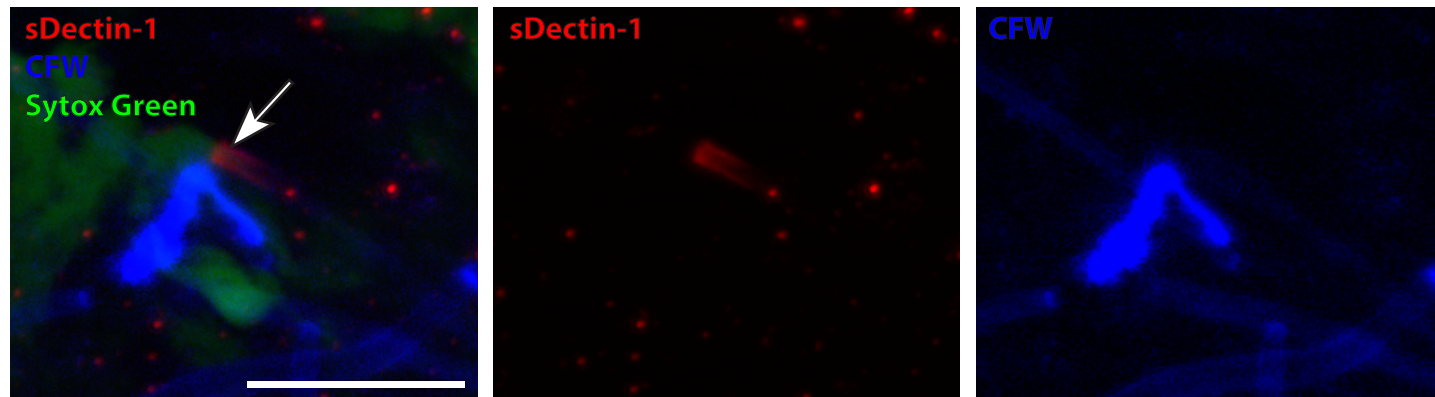

**Figure S2**

Supplement: S2 Fig — Vaginal swab samples were stained as described for Fig 1. In many fields, there were broken ends of filaments that were dead and excluded from analysis. In each of the two representative fields, there is one filament segment that is broken and stained with sDectin-1 (indicated with a white arrow). In panel A, the broken filament end only has sDectin-1 staining on the tip. In panel B, the broken end has sDectin-1 staining descending from the end. Note that in neither case is there enhanced chitin deposition in the cell wall with sDectin-1 staining. Scalebar = 20 μm. Fields are from sample #SP-15980, which is C. albicans. Image is maximum projection of 10 z-slices, created by ImageJ. (PDF) [file pone.0201436.s002.pdf]
